# Carbon Nanomaterials for a Sustainable Future: Advances in Energy Storage and Catalysis

**DOI:** 10.3390/nano15100770

**Published:** 2025-05-21

**Authors:** Yu Ma, Jinchen Fan, Yang Zhao

**Affiliations:** 1 School of Stomatology, Lanzhou University, Lanzhou 730000, China; 2 School of Materials and Chemistry, University of Shanghai for Science and Technology, Shanghai 200093, China; 3 School of Chemistry and Chemical Engineering, Beijing Institute of Technology, Beijing 102488, China

The increasing global demand for sustainable energy and the imperative to address environmental challenges have spurred a renaissance in advanced nanomaterials research [1]. Carbon nanomaterials, including graphene, carbon nanotubes, and various carbon composites, have attracted considerable attention due to their exceptional electrical conductivity, high specific surface areas, and robust chemical stability [2]. These unique attributes enable them to serve as critical components in next-generation energy storage devices and catalytic systems. Their versatile functionality underpins a wide array of applications—from efficient hydrogen evolution [3] and photocatalytic water treatment [4] to smart construction materials and wearable sensors [5]. In this Special Issue, “Carbon Nanomaterials for Green Energy Storage and Catalysis Applications”, we present six contributions—four original research articles followed by two comprehensive reviews—that collectively illustrate recent innovations in the synthesis, characterization, and application of carbon-based materials, offering valuable insights into how these nanomaterials can drive progress toward a more sustainable future.

In one study, Liu and colleagues demonstrate an innovative interface coordination engineering approach to fabricate P–Fe_3_O_4_/Fe@C catalysts derived from an iron-based metal–organic framework [6]. By precisely controlling undercoordinated Fe sites, the catalyst achieves overpotentials of only 160 mV in acidic and 214 mV in alkaline electrolytes at 10 mA·cm^−2^. Such low overpotentials underscore the enhanced electron transfer and water adsorption facilitated by the tailored interfacial structure, offering significant promise for pH-universal water splitting.

Another contribution from Ma, Wu, and colleagues describes a novel synthesis route for embedding Cu nanoparticles into a mesoporous C/SiO_2_ framework [7]. Utilizing a grinding-assisted self-infiltration process followed by in situ reduction, the resulting composite functions as an effective Fenton-like catalyst. Remarkably, it achieves up to 99.9% degradation of tetracycline in a solution with an initial concentration of 500 mg·L^−1^ and maintains high efficiency (94–99% removal) over a wide pH range (3.0–11.0). These data illustrate a practical and environmentally friendly approach to mitigating antibiotic contamination in wastewater.

Addressing the interface between biology and electronics, Alferov and colleagues explore the functionalization of multi-walled carbon nanotubes (MWCNTs) for bio-electrocatalysis [8]. Their strategy involves modifying MWCNT surfaces to immobilize a two-domain bacterial laccase from Catenuloplanes japonicus, thereby facilitating direct electron transfer between the enzyme and the electrode. The reported redox potential of the immobilized enzyme exceeded 500 mV, and the system achieved current densities on the order of hundreds of μA·cm^−2^. These performance metrics provide a solid basis for the further development of enzyme-based biofuel cells.

In a complementary study, Barseghyan and colleagues investigate how the diameter and concentration of MWCNTs affect the mechanical and electrical properties of cement mortars—with and without biosilica [9]. Their systematic experiments revealed that, in 7-day cured samples, the incorporation of MWCNTs increased compressive strength by 6.4% to 10.8% (without biosilica) and by 6.7% to 29.2% (with biosilica), while 28-day compressive strength improvements reached up to 21.7%. Additionally, optimal MWCNT levels (around 0.05 wt.%) resulted in the lowest electrical resistivity and imparted notable piezoresistive behavior, highlighting the potential for smart, self-sensing construction materials.

Complementing this original research, a comprehensive review by Chen and co-workers critically evaluates various synthesis strategies for MnO_2_–carbon materials and their photocatalytic applications in water treatment [10]. Their detailed analysis of structural properties and performance parameters offers valuable insights into the challenges and future prospects of deploying these composites for environmental remediation.

In another review, Zhao and colleagues provide an insightful overview of recent progress in graphene-based wearable temperature sensors [11]. Although the focus is on flexible sensing for health monitoring, their discussion highlights graphene’s exceptional electrical, thermal, and mechanical properties. The review not only elucidates the development of high-performance wearable devices but also suggests potential intersections with energy storage and catalysis.

In conclusion, these contributions underscore the diverse and impactful role of carbon nanomaterials in advancing green energy storage and catalysis. The research articles in this Special Issue not only reveal innovative synthesis routes and practical applications but also deepen our understanding of the fundamental mechanisms governing energy conversion and environmental remediation. Meanwhile, the reviews provide comprehensive analyses of current challenges and outline promising directions for future investigations. Collectively, these studies bridge the gap between laboratory research and real-world application, demonstrating how interdisciplinary approaches can lead to significant breakthroughs in sustainable technologies. We extend our sincere gratitude to all the authors, reviewers, and editors for their invaluable contributions. We are confident that the insights presented in this Special Issue will inspire further collaborative efforts and drive progress toward a more sustainable, resilient, and energy-efficient future.
